# 

**Published:** 1899-12-09

**Authors:** 


### NOTICE TO CORRESPONDENTS.

**All MS., Letters, Books for Review, and other matters intended for the Editor should be addressed The Editor, "The Hospital," The Hospital Building, 28 & 29, Southampton Street, Strand, W.C.**

**The Editor cannot undertake to return rejected MS., even when accompanied by stamped directed envelope.**

**Notice to Advertisers and Subscribers.**

**All Advertisements, Orders for Copies of The Hospital, and Business Communications should be addressed to THE MANAGER (Not to the Editor), The Hospital, The Hospital Building, 28 & 29, Southampton Street, Strand, London, W.C.**

**The Cost of Subscription, post free, is as follows:—Per week, 2½d.; per quarter, 2s. 8d.; per half-year, 5s. 3d.; per annum, 10s. 6d. Foreign:—Per annum, 15s. 2d., payable, in all cases, in advance.**

**NOTICE.—Vol. XXVI. of "The Hospital" (April, 1899, to September, 1899), in handsome cloth binding, is now on sale at the Office of this Journal, price 7s. 6d. Cases for binding the Numbers contained in this Volume 1s. 6d. Index to Vol. XXVI., 2½d., post free.**

**The Monthly Part for December is now ready. Price 6d., by post 8d.**

### BOOKS RECEIVED.

Scientific Press.

"The Nurses' Pocket Diary and Note Book for 1900."

"Surgical Ward Work and Nursing." A Handbook for Nurses and Others. By Alexander Miles, M.D., C.M., F.R.C.S.Edin. Second edition.

Smith, Elder, and Co.

"Health Abroad." A Medical Handbook of Travel by Various Authors. Edited by Edmund Hobhouse, M.D.

### Scraps and Gleanings.

This year's Hospital Saturday Fund collections at Liverpool show an increase of nearly 11¼ per cent. over last year, having amounted to £7,020 odd.

The Chelsea Hospital for Women has received from Mr. J. Passmore Edwards a donation of £105, reducing the debt on the alterations and improvements fund to £1,400.

The honorary surgeons of the Stockport Infirmary are contributing £300 towards the cost of the operating theatre. To meet the cost of all the improvements and extensions a further sum of £4,000 is needed.

The Croydon Hospital Saturday Fund were able to hand over to the Croydon General Hospital a sum of £[illegible]40, being the contribution for this year. The Hospital Saturday Fund has now been established 27 years.

The debt on the building at St. Leonards Hospital still amounts to £209 6s. 9d.

The gross amounts collected and promised up to date for the Clacton and District Cottage Hospital is £3,023 odd, of which sum the handsome amount of £973 odd was obtained through the bazaar.

At a recent meeting of the Committee of the Beckett Hospital, Barnsley, it was resolved, "That this meeting is prepared to sanction the extension of the hospital on the lines laid down in the plans prepared as soon as the £5,000 required for the carrying out is actually collected or promised." These proposed extensions comprise the erection of a children's ward and improved accommodation for the staff of the hospital, providing in all ten additional beds.

### The Student of Medicine.

### APPOINTMENTS.

J. W. Aldred, M.B., Ch B.Vict., appointed House Surgeon to the North Lonsdale Hospital, Barrow-in-Furness. R. D. Boase, L.R.C.P., M.R.C.S.Eng., appointed Medical Officer of Health to the Madron Urban District. P. P. Bradford, L.R.C.P.Lond., M.R.C.S.Eng., appointed Medical Officer for the Bracknell District and Workhouse of the Easthampstead Union. G. P. Chappel, M.D., B.C.Cantab., appointed Honorary Physician to Out-patients at the Tottenham Hospital. G. H. S. Hillyar, L.R.C.P., L.R.C.S.Edin., appointed Medical Officer for the Third District of the Church Stretton Union. J. H. D. Phelps, B.A.Oxon., M.R.C.S., L.R.C.P., appointed House Surgeon to the Buenos Ayres British Hospital. L. Phillips, M.D.Brux., M.R.C.S.Eng., appointed Consulting Surgeon to the Birmingham Hospital for Skin and Urinary Diseases. F. E. Taylor, M.A., M.Sc., M.B., B.Ch., M.R.C.S., L.R.C.P., appointed Senior Resident Medical Officer to Queen Charlotte's Lying in Hospital, London. E. Waggett, M.B., B.C., appointed Surgeon to the London Throat Hospital. F. J. Wethered, M.D., F.R.C.P.Lond., appointed Assistant Physician to the Middlesex Hospital. R. T. Williamson, M.D.Lond., M.R.C.P., appointed Assistant Lecturer in Medicine at Owens College, Manchester. E. E. Willis, L.R.C.P., L.R.C.S.Edin., appointed Medical Officer for the Littleport District of the Ely Union.

St. Thomas's Hospital—House Appointments.—The following gentlemen have been selected as House Officers from Tuesday, December 5th, 1899. House Physicians: F. H. Ellis, B.A., M.B., B.C.Cantab., L.R.C.P., M.R.C.S.; A. H. Greg, B.A., M.B., B.C.Cantab., L.R.C.P., M.R.C.S. (extension); A. Bevan, L.R.C.P., M.R.C.S. (extension); and B. F. Howlett, L.R.C.P., M.R.C.S. Assistant House Physicians: H. R. Beale, L.R.C.P., M.R.C.S.; and L. S. Dudgeon, L.R.C.P., M.R.C.S. House Surgeons: H. J. Phillips, L.R.C.P., M.R.C.S. (extension); P. W. G. Sargent, M.A., M.B., B.C.Cantab., L.R.C.P., M.R.C.S. (extension); S. A. Lucas, L.R.C.P., M.R.C.S. (extension); and H. T. D. Acland, L.R.C.P., M.R.C.S. (extension). Assistant House Surgeons: A. Webb Jones, L.R.C.P., M.R.C.S. (extension); E. A. Gates, L.R.C.P., M.R.C.S. (extension); E. C. Bourdas, L.R.C.P., M.R.C.S. (extension); and N. Unsworth, L.R.C.P., M.R.C.S. (extension). Obstetric House Physicians: (senior) G. B. Thwaites, M.B.Lond., L.R.C.P., M.R.C.S.; and (junior) H. H. R. Clarke, L.R.C.P., M.R.C.S. Clinical Assistants in the Special Department for Diseases of the Throat: A. J. B. Adams, L.R.C.P., M.R.C.S.; and S. H. Belfrage, M.B.Lond. Clinical Assistants in the Special Department for Diseases of the Skin: T. Perrin, L.R.C.P., M.R.C.S. (extension); and Y. Takaki, L.R.C.P., M.R.C.S. Clinical Assistant in the Special Department for Diseases of the Ear: Y. Takaki, L.R.C.P., M.R.C.S. Clinical Assistant in the Electrical Department: E. F. Buzzard, M.A., M.B., B.Ch.Oxon., M.R.C.P.Lond.

### IMPORTANT NURSING MANUALS.

### SURGICAL WARD-WORK AND NURSING.

By ALEXANDER MILES, M.D.Edin., C.M., F.R.C.S.E.

Second Edition. Enlarged and Revised. Demy 8vo., copiously Illustrated, cloth gilt, 3s. 6d. net.

"An illustrated manual which will furnish much needed guidance to the student and the nurse in the early stages of their apprenticeship."—*The Times.*

### NURSING.

By ISABEL ADAMS HAMPTON.

New and Revised Edition. Crown 8vo., 500 pp., Illustrated, cloth gilt, 7s. 6d. net.

"It is not too much to say that in no single volume yet published has the subject been so scientifically carefully, and completely treated as it has been by Miss Hampton. . . ."—*The Hospital.*

### ART OF MASSAGE.

By A. CREIGHTON HALE.

Second Edition. Demy 8vo., Illustrated, cloth gilt, 6s.

"Explains in a series of clearly illustrated articles the many complaints for which massage has been found beneficial, and the special manipulations required in each case."—*Morning Post.*

### PRACTICAL POINTS IN NURSING.

**For Nurses in Private Practice.**

By EMILY A. M. STONEY.

Crown 8vo., Illustrated. 7s. 6d. net.

"The hints given are excellent, and we hope they may be widely read."—*British Medical Journal.*

*Complete Catalogue of Works on Nursing and allied subjects post free on application.*

London: THE SCIENTIFIC PRESS, LTD., 28 & 29, SOUTHAMPTON STREET, STRAND, W.C.

### NOW READY,

A REPRINT OF

### A NURSING HANDBOOK

TOGETHER WITH S. MAW, SON, AND THOMPSON'S

### COMPLETE CATALOGUE OF NURSING REQUISITES.

The above work is now republished at a cost of 1s. per copy. Messrs. S. M., S., and T. have been compelled to make this charge on account of the **extreme popularity** of the work and the great expense incurred in its production.

***Post Free on receipt of 1s.***

### S. MAW, SON, and THOMPSON,

7 to 12, ALDERSGATE STREET, LONDON, E.C.

